# Activation of γ-globin expression by LncRNA-mediated *ERF* promoter hypermethylation in β-thalassemia

**DOI:** 10.1186/s13148-023-01614-6

**Published:** 2024-01-13

**Authors:** Xiuqin Bao, Yuanyi Gao, Zhongju Wang, Yuhua Ye, Diyu Chen, Yangjin Zuo, Cunyou Zhao, Xiangmin Xu

**Affiliations:** 1 Medical Genetic Center, Guangdong Women and Children Hospital, Guangzhou, 514000 Guangdong China; 2 Innovation Center for Diagnostics and Treatment of Thalassemia, Nanfang Hospital, Southern Medical University, Guangzhou, 510515 Guangdong China; 3 Department of Medical Genetics, School of Basic Medical Sciences, Southern Medical University, Guangzhou, 510515 Guangdong China; 4 Guangdong Engineering and Technology Research Center for Molecular Diagnostics of Human Genetic Diseases, Guangzhou, 510515 Guangdong China; 5 Maternal and Children Metabolic-Genetic Key Laboratory, Guangdong Women and Children Hospital, Guangzhou, 514000 Guangdong China; 6 Key Laboratory of Mental Health of the Ministry of Education, Guangdong-Hong Kong-Macao Greater Bay Area Center for Brain Science and Brain-Inspired Intelligence, and Guangdong Province Key Laboratory of Psychiatric Disorders, Southern Medical University, Guangzhou, China

**Keywords:** *RP11-196G18.23*, Fetal hemoglobin, *ERF* promoter hypermethylation, β-Thalassemia, γ-Globin

## Abstract

The mechanism that drives the switch from fetal to adult hemoglobin (Hb) provides a therapeutic target for β-thalassemia. We have previously identified that hypermethylation of transcription factor *ERF* promoter reactivated γ-globin expression. To uncover the mechanism underlying the hypermethylation of *ERF* promoter, we performed RNA sequencing in β^0^/β^0^-thalassemia patients and identified an upregulated long noncoding RNA (*RP11-196G18.23*) associated with HbF production. *RP11-196G18.23* bound to the *ERF* promoter and recruited DNA methyltransferase 3A to promote DNA hypermethylation-mediated ERF downregulation, thereby ameliorating ERF-induced γ-globin inactivation. The identification of *RP11-196G18.23* provides an epigenetic mechanism for the reactivation of fetal γ-globin expression for β-hemoglobinopathies.

**Supplementary Information:**

The online version contains supplementary material available at 10.1186/s13148-023-01614-6.

## Introduction

Reactivating fetal hemoglobin (HbF, α_2_γ_2_) holds a therapeutic target for β-thalassemia and sickle cell disease. Several modulators, such as transcription factors (TFs) BCL11A and LRF, have been uncovered to regulate HbF expression by directly binding to γ-globin promoter [1, 2]. Our previous study also identified the transcription factor ERF as a repressor of HbF that binds to two regulated elements—one located 3.5 kb upstream of *HBG2* and the other 1.5 kb downstream of *HBG1* [3]*.* We found that the hypermethylation-mediated transcriptional inactivation of ERF can reproduce γ-globin in high HbF β-thalassemia patients. However, the molecular mechanism underlying the hypermethylation of the *ERF* prompter in high HbF patients remains unclear. Recently, long noncoding RNAs (lncRNAs) have emerged as critical regulators of gene expression, performing functions in cis or in trans. Such regulators have already been shown to play regulatory roles in normal erythropoiesis and disease conditions, including erythroid cell survival, heme metabolism, globin switching and regulation, etc [4–6]. For example, a lncRNA transcribed from the pseudogene *HBBP1* locus interacts with the TF ELK1 to regulate the expression of γ-globin gene [7]. In addition, HMI-LNCRNA transcribed by *MYB* enhancer region can inhibit HbF expression and delay erythroid maturation [8], but the specific mechanism is not clear. These studies indicate that lncRNAs are involved in the regulation of γ-globin expression, but the mechanism by which lncRNAs regulate γ-globin expression through TFs interaction needs to be further studied. Here, we performed strand-specific RNA-seq analysis of bone marrow (BM)-derived GYPA^+^ erythroid cells from 6 β^0^/β^0^-thalassemia patients who were stratified into low- (HbF_L_: 0.1–0.4 g/dL,* n* = 3) and high HbF (HbF_H_: 8.9–9.2 g/dL, *n* = 3) expression groups used in our previous study [3] to screen for differentially expressed lncRNAs (DE-lncRNAs) associated with HbF production and their participation in regulating ERF expression.

## Materials and methods

### Patients and RNA sequencing (RNA-seq)

RNA-seq analysis was performed based on the patients in our previous study [3]. These patients were divided into two group (HbF_H_: 8.9–9.2 g/dL, and HbF_L_: 0.1–0.4 g/dL) based on the HbF level. All patients gave the informed consent (Additional file 1). Differentially expressed lncRNAs (DE-lncRNAs) between HbF_H_ and HbF_L_ groups of β^0^/β^0^-thalassemia patients were screened according to the following criteria: |log2FoldChange|> 0.5 and probability ≥ 0.8. More details were provided in the Additional file 1.

### In vitro validation

Coding potential ability of lncRNA *RP11-196G18.23* was performed using open reading frame finder from NCBI and phyloSCF in silico prediction. Subcellular localization of *RP11-196G18.23* in the HUDEP-2 cell line was identified using Fluorescence in situ hybridization (FISH). Chromatin isolation by RNA purification (ChIRP), RNA immunoblotting and RNA immunoprecipitation were employed to detect the interaction among *RP11-196G18.23*, *ERF* promoter and DNA methyltransferases DNMT1and DNMT3A. Chromatin immunoprecipitation (ChIP) was performed to investigate the enrichment of DNMT3A to *ERF* promoter. CRISPR/Cas9 system was used to delete the binding sequences of *RP11-196G18.23* on *ERF* promoter. Bisulfite sequencing was performed to detect methylation level of CpG sites in *ERF* promoter. More details were descried in the Additional file 1. A two-tailed Student’s *t* test and ANOVA from SPSS v.20 software were used for comparisons between the indicated groups studied. *p* values of less than 0.05 were considered to be statistically significant.

## Results and discussion

**Fig. 1:**
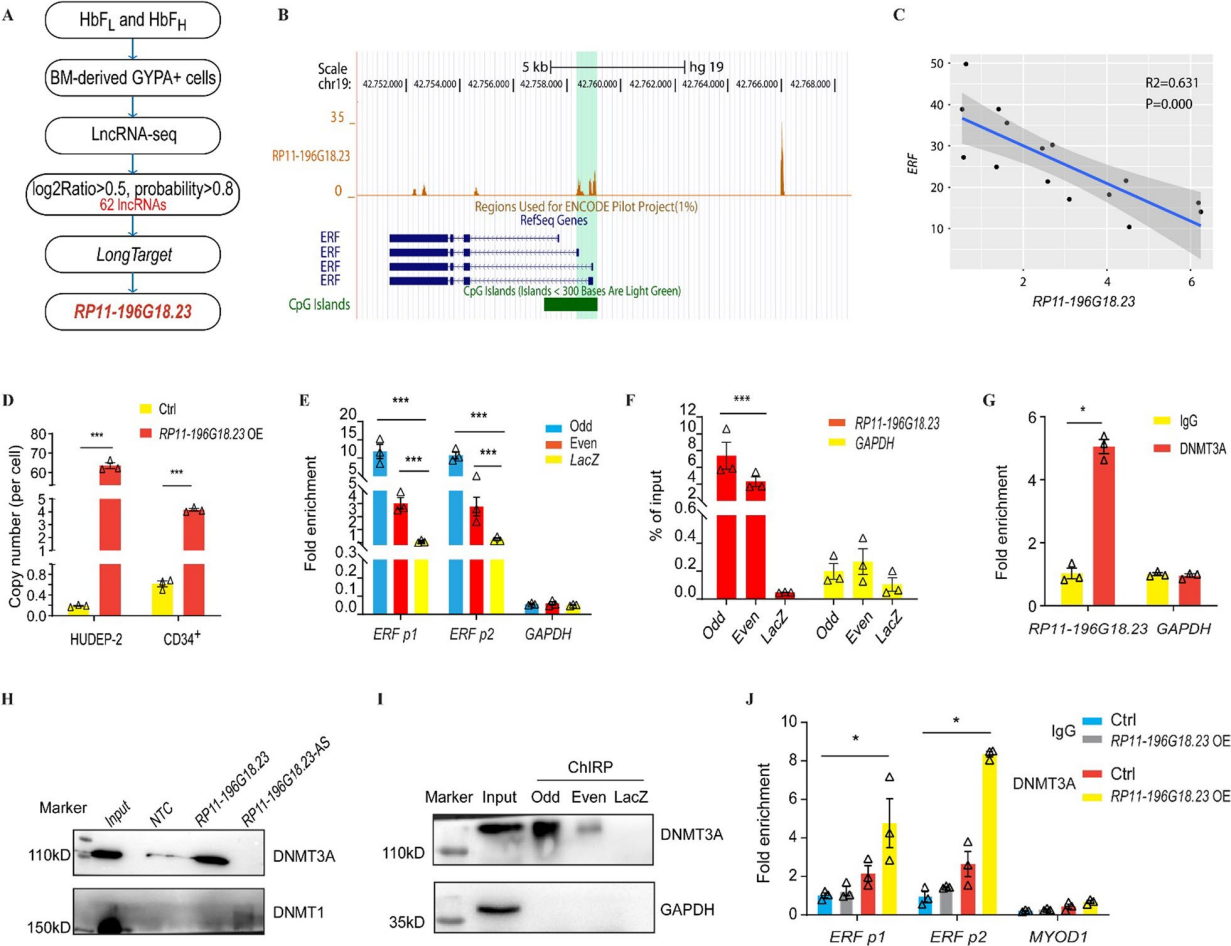
LncRNA *RP11-196G18.23* binds to *ERF* promoter and interacts with DNMT3A. **A** Flowchart of DE-lncRNAs analysis and candidate lncRNAs screening. DE-lncRNAs were analyzed according to log2Ratio > 0.5 and probability > 0.8. LongTarget was used to predict the interactive lncRNAs on *ERF* promoter. **B** *RP11-196G18.23* was predicted to bind to the *ERF* promoter shown in the UCSC genome browser. The orange peaks show the binding region of *RP11-196G18.23* in the *ERF* promoter. The number above ‘0’ indicates the maximal number of overlapping triplexes at an address in the region. The shadowed light green bar marks the lncRNA binding sites in the promoter regions. **C** Regression analysis between *ERF* and *RP11-196G18.23* expression based on the data from GEO database (GSE53983). The gray region indicated the 95% confidence interval. **D** Copy number of *RP11-196G18.23* overexpression in HUDEP-2 and CD34^+^ HSPCs. **E**, **F** ChIRP analysis of *RP11-196G18.23* interaction with *ERF* in *RP11-196G18.23 OE* HUDEP-2 cells. The retrieved *ERF* DNA (**E**) and RNA (**F**) was quantified by qPCR. *LacZ*, negative control probe. Odd and even, the RP11-196G18.23 probes. ERF p1 and p2, two fragments on *ER*F promoter. *GAPDH*, negative control for qPCR. **G** RIP analysis of interaction of *RP11-196G18.23* with DNMT3A in HUDEP-2 cells. IgG, the control for the specificity of the anti-DNMT3A antibody. *GAPDH*, the negative control. **H** RNA pull-down analysis of specific association of DNMT1 or DNMT3A with lncRNA *RP11-196G18.23* in *RP11-196G18.23* OE HUDEP-2 cells. Non-template control (NTC), negative control. AS, antisense sequence of *RP11-196G18.23*. **I** Western blot analysis of the protein retrieved from *RP11-196G18.23*-ChIRP. **J** ChIP analysis of association of DNMT3A with the *ERF* promoter (p1 and p2 region) was performed using HUDEP-2 cells without (Ctrl) or with *RP11-196G18.23* OE. *MYOD1*, negative control for qPCR. Data are shown as the means ± SEM from at least two independent experiments (*p < 0.05; ***p < 0.001)

We analyzed the DE-lncRNAs between HbF_H_ and HbF_L_ groups and identified 62 lncRNAs that showed significant, HbF production-associated alterations. Among them, an HbF-associated upregulated lncRNA *RP11-196G18.23* (LogFC = 0.5 and probability = 0.8; Fig. 1A, Additional file 1: Table S1) was predicted to have binding sites in the *ERF* promoter region using the LongTarget tool (Fig. 1B). We then validated the expression of *RP11-196G18.23* in the β^0^/β^0^-thalassemia patients using real-time quantitative reversely transcribed PCR (qRT-PCR) and confirmed that *RP11-196G18.23* expression in the high HbF group was approximately three times higher than that of the low HbF group (Additional file 1: Fig. S1A). To determine the relationship between *RP11-196G18.23* and *ERF*, we first characterized the protein coding ability and subcellular localization of *RP11-196G18.23*. *RP11-196G18.23* had no protein coding ability, as predicted by in silico analysis (Additional file 1: Fig. S1B) and an in vitro experiment involving the fusion of open reading frame (ORF) and EGFP (Additional file 1: Fig. S1C–E), and it was predominantly expressed in the nucleus, as determined by fluorescence in situ hybridization (FISH) (Additional file 1: Fig. S1F). We then analyzed the public RNA-sequencing data (GSE53983) of CD34^+^ HSPCs from Gene Expression Omnibus database (GEO) and observed a negative correlation between *ERF* and *RP11-196G18.23* (Fig. 1C). LncRNAs are reported to repress genes by binding to the promoter and recruit DNMTs to mediate the DNA methylation of target regions [9]. In addition, our previously study [3] demonstrated that DNMT3A participates the regulation of ERF. Thus, we hypothesized that *RP11-196G18.23* might mediate *ERF* hypermethylation and downregulation by binding to its promoter, which could be involved in reactivation of *HBG* expression. To validate whether *RP11-196G18.23* could inhibit *ERF* expression by binding to its promoter, we performed ChIRP analysis using *RP11-196G18.23* overexpressed HUDEP-2 cell lysates (Fig. 1D). We observed that *RP11-196G18.23* could bind to *ERF*, as demonstrated by qPCR analysis of DNA (Fig. 1E) or RNA (Fig. 1F) retrieved from *RP11-196G18.23*-ChIRP. We then carried out immunoblotting analysis using anti-DNMT3A and DNMT1 antibodies in HUDEP-2 cells. We observed that *RP11-196G18.23* could bind to DNMT3A but not DNMT1 (Fig. 1G, H). Protein retrieved from *RP11-196G18.23*-ChIRP also confirmed this result (Fig. 1I). ChIP-qPCR analysis of the HUDEP-2 cell lysate confirmed that *RP11-196G18.23* enhanced the recruitment of DNMT3A to the *ERF* promoter region (Fig. 1J). These data indicate that lncRNA *RP11-196G18.23* could bind to *ERF* promoter and interact with DNMT3A.

**Fig. 2:**
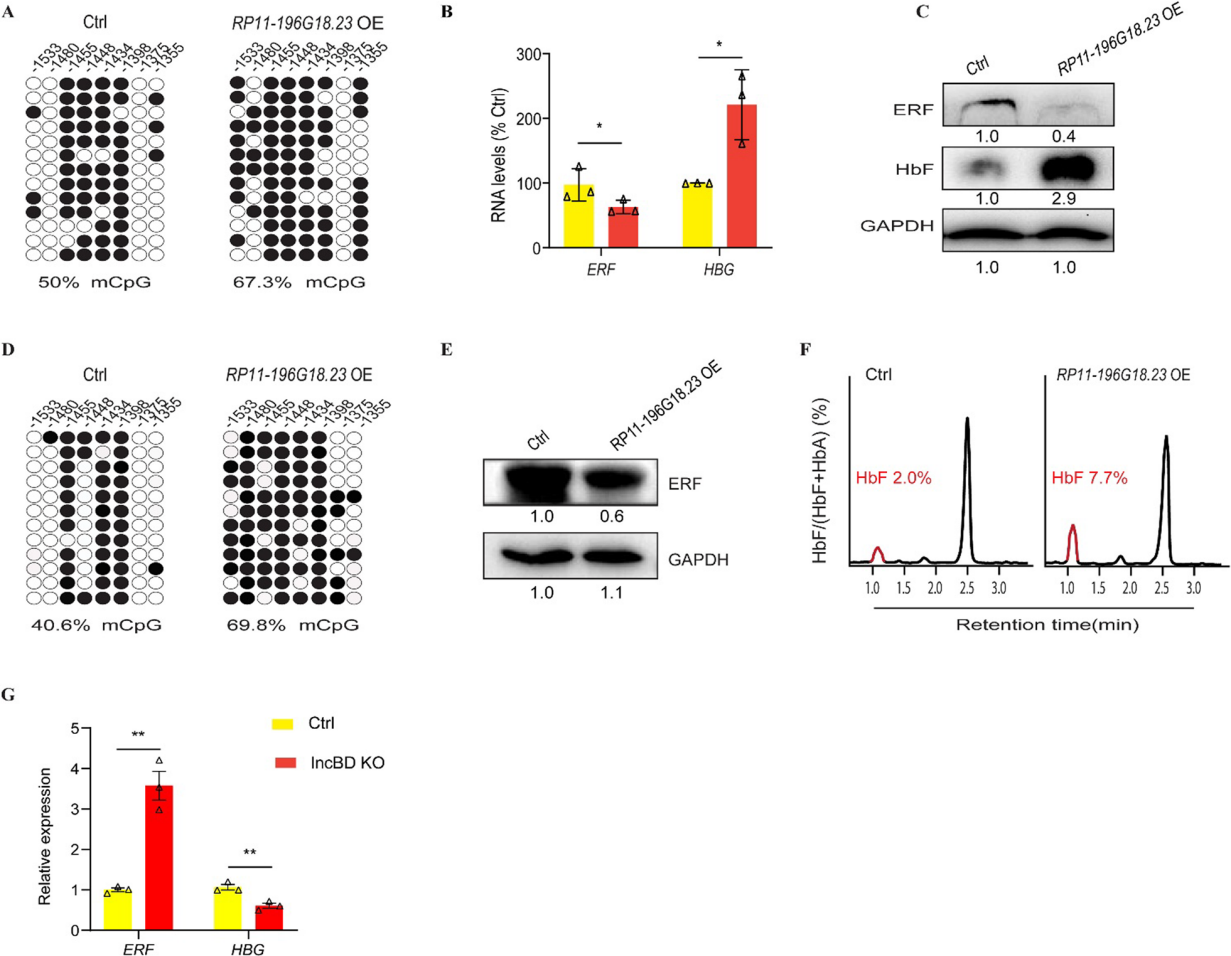
LncRNA *RP11-196G18.23* mediates *ERF* promoter hypermethylation and leads to reactivation of γ-globin. **A** The *ERF* promoter methylation level examined by clone-seq in HUDEP-2. Each row of eight CpG sites within a group represents a single bisulfite-treated clone with methylated CpGs (●) or unmethylated CpGs (○). **B**, **C** The *ERF* and γ-globin mRNA and protein levels were examined by qPCR (**B**) or Western blotting (**C**) in *RP11-196G18.23* OE HUDEP-2 cells. The band intensities measured by ImageJ were showed underneath each panel. **D** The ERF promoter methylation level in CD34^+^ HSPCs. **E, F** The ERF protein level examined by Western blot (**E**) and the Hb F production examined by HPLC **(F**) in CD34^+^ HSPCs. **G** qPCR analysis of *ERF* and γ-globin mRNA level in wild type (Ctrl) and RP11-196G18.23 binding sequences disrupted HUDEP-2 cells. Data are shown as the means ± SEM from at least two independent experiments (*p < 0.05; ***p < 0.001)

In the nucleus, lncRNAs regulate gene expression by controlling the local chromatin structure or recruiting regulatory molecules to specific loci. A lncRNA *BGLT3* has been reported to regulate γ-globin transcription both in cis and in trans [6]. In cis, BGLT3 gene locus transcriptionally activates fetal γ-globin genes via facilitating chromatin looping between LCR and γ-globin promoters [4]. In trans, BGLT3 interacts with the Mediator complex, such as MED12 on chromatin to aid γ-globin transcriptional assembly [10]. Therefore, we wonder if *RP11-196G18.23* could mediate the *ERF* promoter hypermethylation by recruiting DNMT3A and leads to reactivation of γ-globin. To validate this hypothesis, we overexpressed *RP11-196G18.23* in HUDEP-2 cells and CD34^+^ HSPCs (Fig. 1D). We observed that the methylation level of *ERF* promoter was significantly increased, while the endogenous *ERF* mRNA and protein levels were decreased both in HUDEP-2 cells (Fig. 2A–C) and CD34^+^ HSPCs (Fig. 2D, E). DNMT3A also enriched in the *ERF* promoter after *RP11-196G18.23* overexpression (Fig. 1I). More importantly, we observed that *RP11-196G18.23* overexpression could stimulate γ-globin mRNA and protein levels in both HUDEP-2 cells (2.1-fold change relative to the control) (Fig. 2B, C) and CD34^+^ HSPCs (7.7% of total hemoglobin in *RP11-196G18.23* overexpression CD34^+^ HSPCs, compared with 2.0% in control cells) (Fig. 2F). However, compared with the effect of major regulators such as BCL11A and LRF on the level of HbF, the effect of *RP11-196G18.23* overexpression on γ-globin reactivation is modest. This is probably due to the indirect effect of *RP11-196G18.23* in regulating γ-globin rather than direct binding to the *HBG* promoter. In addition, there may be some other complexes binding to *ERF* promoter remain to be uncovered. To further determine the association between *RP11-196G18.23* and *ERF* promoter, we disrupted the binding sequences of *RP11-196G18.23* on *ERF* promoter and found that the expression of ERF was significantly increased while the γ-globin was decreased (Fig. 2G and Additional file 1: Figure S2). Our previously study [3] also demonstrated that the expression of ERF was decreased after hypermethylation on *ERF* promoter by site-specific methylation through dCas9-MQ1-sgRNA system, and consequently, the γ-globin expression was increased. Altogether, these results demonstrated that *RP11-196G18.23* could inhibit *ERF* gene expression to reactivate *HBG* expression by recruiting DNMT3A and enhancing *ERF* methylation (Additional file 1: Figure S3).

In conclusion, our study demonstrated that *RP11-196G18.23* bound to *ERF* promoter and then recruited DNMT3A to mediate hypermethylation of *ERF* promoter, resulting in downregulation of *ERF* and upregulation of γ-globin in patients with high HbF. Our research provides an epigenetic mechanism for the reactivation of fetal γ-globin expression.

### Supplementary Information

**Additional file 1**. Materials and methods, tables and figures.

## Data Availability

All the data were showed through the whole manuscript and Additional file 1. Public data (GSE53983) were available in Gene Expression Omnibus database (GEO).
