# Supplementary material for: Activation of γ-globin expression by LncRNA-mediated ERF promoter hypermethylation in β-thalassemia
Source: Clin Epigenetics. 2024 Jan 13;16:12. doi: 10.1186/s13148-023-01614-6 (PMC10787479; doi:10.1186/s13148-023-01614-6)
Supplement: Supplementary file 1 — Additional file 1. Materials and methods, tables and figures. [file 13148_2023_1614_MOESM1_ESM.docx]

**Supplementary appendix**

**Methods and materials**

**RNA-seq and data analysis.** We performed lncRNA-seq using the β^0^/β^0^-thalassemia individuals used in our previous study^1^, who were divided into two groups (low HbF: HbF_L_ and high HbF: HbF_H_) based on the HbF level. Total RNA was extracted from GYPA positive cells derived from bone marrow. mRNA and non-coding RNAs are enriched by removing rRNA from the total RNA with kit. By using the fragmentation buffer, the mRNAs and non-coding RNAs are fragmented into short fragments (about 200~500nt), then the first-strand cDNA is synthesized by random hexamer-primer using the fragments as templates, and dTTP is substituted by dUTP during the synthesis of the second strand. Short fragments are purified and resolved with EB buffer for end reparation and single nucleotide A (adenine) addition. After that, the short fragments are connected with adapters, then the second strand is degraded using UNG(Uracil-N-Glycosylase) finally. After agarose gel electrophoresis, the suitable fragments are selected for the PCR amplification as templates. During the QC steps, Agilent 2100 Bioanaylzer and ABI StepOnePlus Real-Time PCR System are used in quantification and qualification of the sample library. At last, the library was sequenced using Illumina HiSeqTM 2000. Sequenced reads were aligned to the UCSC hg19 (human) genome using BWA and Bowtie, and the coding gene and isoform expression levels for mRNA and noncoding gene level for lncRNA were quantified by the RNA-Seq by Expectation Maximization (RSEM) package. Differential expressed (DE) lncRNA analysis was carried out using the NOISeq package. We screened DE-lncRNAs according to the following criteria: |log2FC| > 0.5 and probability ≥ 0.8 to identify 62 DE-lncRNAs between the HbF_H_ and HbF_L_ groups.

**Characterization of lncRNA *RP11-196G18.23.*** We performed a coding potential examination using open reading frame finder from NCBI^2^ and phyloSCF^3^ *in silico* prediction, and an *in vivo* experiment with ORF-fused GFP cDNA in pGEFP-N1 vector transfected in HEK293T cells. We further examined the copy number of *RP11-196G18.23* transcript per cell by a standard curve qPCR as described.^4^ We then employed Fluorescence *in situ* hybridization (FISH) to identify the subcellular localization of *RP11-196G18.23* in the HUDEP-2 cell line using the Ribo Fluorescence *In Situ* Hybridization Kit (C10910, RiboBio, Guangzhou, China). Cells were washed with PBS twice and were then fixed in 4% paraformaldehyde for 10 minutes. PBS containing 0.5% Triton X-100 was then used to destroy the membrane. Cells were washed in PBS three times. Cell suspensions were added dropwise to the cover slip. Prehybridization solution was added to the cells and incubated at 37°C for 30 minutes. The prehybridization solution was subsequently removed, and 100 µl of hybridization solution containing 2.5 µl of 20 µM probes was added to the cells and incubated at 37°C overnight. After sequential washes in 4× SSC/2× SSC/1× SSC solution, DAPI diluted with PBS (1:100) was added to stain nuclei. After three washes with PBS, an anti-fluorescence quencher was employed for mounting purposes. Confocal microscopy was used to assess localization.

**Chromatin immunoprecipitation (ChIP).** ChIP assays were performed with cell extracts from HUDEP-2 cells using anti-DNMT3A (ab307503 Abcam) antibody as recommended (EZ-ChIP, Merck Millipore, Germany). A total of 1×10^7^ cells were fixed with 1% formaldehyde at 37°C for 10 minutes, and the reaction was terminated by the addition of glycine. Sonication was performed using a Covaris S2 instrument (Covaris). The chromatin solution was first incubated with 60 µl of Protein A/G Dynabeads (Merck Millipore) at 4°C for 1 hour on a rotating shaker to prevent nonspecific binding. ChIP was then performed using 2 µg of antibody at 4°C overnight on a rotating shaker. Antibody/protein complexes were collected following incubation with 60 µl of Protein A/G Dynabeads at 4°C for 2 hours. Beads were sequentially washed once each with the following buffers: low-salt buffer, high-salt buffer, LiCl buffer and Tris-EDTA (TE) buffer. Complexes were then eluted from the beads in buffer containing 50 mM Tris-HCl (pH 8.0), 1% SDS, and 10 mM EDTA. After reverse crosslinking (at 65°C overnight) and proteinase K treatment, DNA was extracted (Merck Millipore) for further high-throughput sequencing or for qPCR analysis of target genes.

ChIP-qPCR was performed in a Bio-Rad real-time qPCR system with SYBR. *ETS2* pro served as the positive control, while *MYOD1* served as the negative control. The relative enrichment of DNMT3A on ERF promoter was determined relative to the enrichment of the IgG. The primers used in qPCR were listed in Table S2 in the supplementary appendix.

**RNA immunoblot.** We examined the interaction between lncRNA *RP11-196G18.23* and DNMT3A using *in vitro* transcribed biotin-labeled lncRNAs. Magnetic streptavidin-coated beads (S1420s, New England Biolabs) were incubated with either sense (test) or antisense (control) biotinylated transcript (10 µg), which was transcribed and labeled *in vitro* per the instructions of the RNAmax-T7 biotin-labeled transcription kit (C11002, RiboBio, Guangzhou, China), at RT for 20 minutes. After 3 washes with 1× binding/washing buffer (containing an RNase inhibitor), HEK293T cell lysates were added to the beads and incubated with rotation in 4°C for 2 hours. Next, 400 μl of cell lysis buffer A containing an RNase inhibitor and proteinase inhibitor cocktail were added and washed 5 times. The bead-RNA-protein complexes were eluted with 0.1% SDS and analyzed by immuno blotting using anti-DNMT1 (ab2850, Abcam) and anti-DNMT3A antibodies (ab13537, Abcam).

**RNA immunoprecipitation.** We further examined interaction between lncRNA *RP11-196G18.23* and DNMT3A in HUDEP-2 cells as previously described.^5^ Cells (1-2×10^7^) were harvested and washed twice with PBS. A 1× polysome lysis buffer was used to resuspend and lyse the cells. Protein G-coated magnetic beads were prepared by washing with 0.5 ml of NT-2 buffer twice and incubation with 5 µg of anti-DNMT3A antibody (ab2850, Abcam, UK) and normal rabbit IgG separately at RT for 1 hour. The antibody-bead complexes were washed six times with NT-2 buffer and resuspended in 900 μl of NT-2 buffer. Cell lysates (100 μl) were added to each antibody-bead reaction and incubated overnight at 4°C. After overnight incubation, the tubes were placed on the magnetic support in ice, and the supernatant was discarded. RNA was extracted by using phenol-chloroform. qRT-PCR analysis of *RP11-196G18.23* from retrieved RNAs was used to analyze the interaction between lncRNA *RP11-196G18.23* and DNMT3A.

**Chromatin isolation by RNA purification (ChIRP).** We performed ChIRP assay to identify interaction between lncRNA *RP11-196G18.23* and the *ERF* promoter region in *RP11-196G18.23* OE HUDEP-2 cells using Chromatin Isolation by RNA Purification kit (Bers5104, BersinBio, Guangzhou, China). Briefly, cultured cells were crosslinked *in vivo* and lysed with swelling buffer and nuclear lysis buffer in a homogenizer. Cell lysates were then subjected to sonication. The size of the DNA fragments should be 100 bp to 500 bp. After sonication, 100 μl of agarose beads was added to the precleared lysate. 13 antisense oligo probes were designed and synthesized by RiboBio. Then, 1 ml of hybridization buffer and 10 μl of protease inhibitor, as well as DTT and 2 μl of RNase inhibitor were added and incubated at 65°C for 10 minutes. Then, 8 μl of probe was added to each tube and incubated at 37°C for 30 minutes, 50°C for 5 minutes and 37°C for 3 hours, successively. In addition, 100 μl of streptavidin beads per sample were prepared by washing with 10 mM Tris-HCl (pH 7.5) 5 times and collected onto a magnetic rack and resuspended in hybridization buffer. After incubation, streptavidin beads were added and incubated at RT for 30 minutes. Beads were collected and washed 5 times with 1 ml of wash buffer. Finally, 1 ml of wash buffer was added to resuspend the beads, and the mixture was then divided into 2 tubes of 200 µl and 800 µl for RNA and DNA elution, respectively. For RNA elution, 5× proteinase K buffer and proteinase K were added and incubated at 55°C for 1 hour. Phenol-chloroform was used to isolate RNA. A 2 μl of isolated RNA per well was used for qRT-PCR analysis of the retrieved lncRNA *RP11-196G18.23*. For DNA elution, 150 μl of DNA elution buffer and 1.5 μl of RNase A were added and incubated at 37°C for 30 minutes. Proteinase K (5 μl) was added and incubated at 55°C for 1 hour. Phenol-chloroform was used to isolate DNA. 2 μl of isolated DNA per well were used for qPCR analysis of the retrieved *ERF* promoter DNA fragment. *GAPDH* served as the negative control.

**Cell culture and differentiation.** HUDEP-2 and CD34^+^ erythroid progenitor cells were culture as previous described. Briefly, HUDEP-2 cells were maintained in StemSpan SFEM medium (09650, Stem Cell Technologies, Canada) supplemented with human stem cell factor (hSCF, 50 ng/ml, P21583.1, R&D systems, Germany), erythropoietin (EPO, 3 IU/ml, KIRIN, Japan), dexamethasone (DEX, 10^-6^ M, D2915, Sigma) and doxycycline (Dox, 1 µg/ml, 631311, Sigma).^6^ Granulocyte colony-stimulating factor (G-CSF)-mobilized adult human CD34^+^ erythroid progenitor cells were isolated from the peripheral blood of normal individuals and separated by a CD34 microbead kit (Miltenyi Biotec, Germany). CD34^+^ erythroid progenitor cells were cultured in StemSpan SFEM basic medium supplemented with FBS (10%), EPO (1 IU/ml), hSCF (50 ng/ml) and interleukin-3 (IL-3, 10 ng/ml, Stem Cell Technologies, Canada) for 6 days. On day 6, the medium was replaced with SFEM supplemented with FBS (30%) and EPO (3 IU/ml), and cells were cultured for an additional 10 days.^7^

***RP11-196G18.23* overexpression (OE).** To construct the OE vector, *RP11-196G18.23* was amplified by polymerase chain reaction (PCR). The PCR product was ligated into pHAGE-EF1α-MCS-IZsGreen vector. OE lentivirus was constructed by co-transfecting pHAGE-EF1α-MCS-IZsGreen plasmid with pMD2G and psPAX2 into HEK293T cells. Transduction of viral particles into HUDEP-2 and CD34^+^ HSPCs (after four days of culture) was performed according to the manufacturer’s protocol. After 72 hours of transduction, transduced cells were selected by puromycin (1 μg/ml). OE of *RP11-196G18.23* was confirmed by qRT-PCR.

**Disrupting the binding sequences of *RP11-196G18.23* on *ERF* promoter.** To delete the binding sequences of *RP11-196G18.23*, we designed sgRNA and cloned into lentiCRISPRv2 vector. Transfection was the same as previously described. Lentivirus were produced using 293T and transfected into HUDEP-2 cells. After transfection of 3 days, 1 μg/ml puromycin were added to select the positive cells for 3 days. After six days of culture and selection, cells were harvested to extract DNA. We performed sanger sequencing to confirm the deletion. sgRNAs were listed in **Table S2**.

**qRT-PCR.** Total RNA was extracted using trizol (ThermoFisher Scientific, cat #15596018). cDNAs were prepared by reverse transcription using Hifair III 1^st^ Strand cDNA synthesis Suppermix (YEASEN, cat #11141ES60). qPCR reaction was prepared with Hieff qPCR SYBR Green (YEASEN, cat #11201ES08) and run on Roche Real time qPCR machine.

**Western blot.** Western Blot was performed as standard protocol. The primary antibodies were as follow: anti-HbF (ab137096, Abcam), anti-GAPHD (129-10312, Ray antibody), and anti-ERF (ab153726, Abcam) antibodies.

**High performance liquid chromatography (HPLC)**. 1×10^7^ cells were harvested and 100 μl HPLC-grade water was added to lysis. Incubated on ice for 10 minutes and then subjected to 3 freeze-thaw cycle. HbF level was calculated by Variant II (Bio-Rad Laboratories, USA).

**Bisulfite cloning.** The methylation level of the *ERF* promoter region was further determined using bisulfite cloning method. Bisulfite modification of genomic DNA was performed with [EpiArtTM DNA Methylation Bisulfite Kit](http://www.casmart.com.cn/product-details/page/545/212827826) (EM101, Vazyme, China). Bisulfite-treated DNA were purified according to the manufacturer’s protocols and diluted with a final volume of 10 µl. Nested PCR were performed using 2 µl bisulfite-treated DNA as template. Bisulfite cloning was performed by cloning the nested PCR products into the pMD19T cloning vector (D104, TAKARA, Japan) and sequencing analysis of 10 to 15 clones. Primers used for nested PCR was showed in Table S2.

**References**

1. Bao, X. *et al.* Epigenetic inactivation of ERF reactivates gamma-globin expression in beta-thalassemia. *Am J Hum Genet* **108**, 709-721 (2021).

2. Nishikawa, T., Ota, T. & Isogai, T. Prediction whether a human cDNA sequence contains initiation codon by combining statistical information and similarity with protein sequences. *Bioinformatics (Oxford, England)* **16**, 960-7 (2000).

3. Lin, M.F., Jungreis, I. & Kellis, M. PhyloCSF: a comparative genomics method to distinguish protein coding and non-coding regions. *Bioinformatics* **27**, I275-I282 (2011).

4. Chen, J.J. Regulation of protein synthesis by the heme-regulated eIF2alpha kinase: relevance to anemias. *Blood* **109**, 2693-9 (2007).

5. Gagliardi, M. & Matarazzo, M.R. RIP: RNA Immunoprecipitation. *Methods in molecular biology (Clifton, N J )* **1480**, 73-86 (2016).

6. Kurita, R. *et al.* Establishment of Immortalized Human Erythroid Progenitor Cell Lines Able to Produce Enucleated Red Blood Cells. *Plos One* **8**, e59890 (2013).

7. Sun, Z.W. *et al.* miR-150 inhibits terminal erythroid proliferation and differentiation. *Oncotarget* **6**, 43033-43047 (2015).

**Table S1. The 62 lncRNAs.**

| gene_id | locus | log2Ratio(HbFH/HbFL) | probability |
| --- | --- | --- | --- |
| 3043 | chr11:5246695-5248301 | 21.04709506 | 0.998941341 |
| 9663 | chr18:2916991-3011945 | 13.86527769 | 0.945807869 |
| 1277 | chr17:48261456-48279000 | 13.47703429 | 0.931187142 |
| n334786 | chr10:44865604-44880545 | 13.16099538 | 0.916397992 |
| 3107 | chr6:31236528-31239855 | 12.62130672 | 0.885496367 |
| n343048 | chrX:73012039-73072588 | 12.51074769 | 0.878278235 |
| XLOC_000180\|- | chr1:14275754-14275965 | 12.48269873 | 0.876297259 |
| n371509 | chrX:73012039-73072588 | 12.04393865 | 0.84191489 |
| XLOC_024675\|n324798 | chr21:36160097-36421595 | 12.01487981 | 0.839637169 |
| XLOC_020595\|- | chr2:102721471-102721689 | 11.98730665 | 0.837407568 |
| XLOC_000187\|- | chr1:14381892-14382124 | 11.94343056 | 0.833509777 |
| 349136 | chr7:151077559-151112174 | 11.89839287 | 0.829275139 |
| 1307 | chr1:32117847-32169768 | 11.83697641 | 0.823653016 |
| 1291 | chr21:47401662-47424963 | 11.82981212 | 0.822979324 |
| 4854 | chr19:15050245-16770968 | 11.78527597 | 0.819330157 |
| 1278 | chr7:94023872-94060544 | 6.208690565 | 0.820661502 |
| n335533 | chr5:40831429-40835387 | 4.008924048 | 0.829162857 |
| 3050 | chr16:202853-204504 | 3.684807518 | 0.818688546 |
| n410076 | chr19:13945329-13947103 | 3.61904966 | 0.802880837 |
| 439996 | chr10:91137812-91144962 | 2.975445559 | 0.847416711 |
| 10124 | chr7:12726451-12730559 | 1.880430878 | 0.810307492 |
| 3310 | chr1:161494035-161497073 | 1.878649805 | 0.801902379 |
| 9911 | chr1:205197037-205242471 | 1.813989325 | 0.809000209 |
| RP11-196G18.23 | chr1:149802876-149804109 | 0.704315447 | 0.815498623 |
| 6280 | chr1:153330329-153333503 | -1.727596675 | 0.803273824 |
| 9168 | chr2:85132762-85133799 | -1.76372427 | 0.80467735 |
| 713 | chr1:22979681-22988029 | -1.850899754 | 0.800129926 |
| 1509 | chr11:1773981-1785222 | -1.925354626 | 0.813796256 |
| 6414 | chr5:42756919-42812024 | -1.983195729 | 0.81237669 |
| 341 | chr19:45417576-45422606 | -2.021575948 | 0.817934652 |
| 3162 | chr22:35777059-35790207 | -2.059621024 | 0.815352165 |
| 3988 | chr10:90973325-91011660 | -2.135671272 | 0.810123029 |
| 967 | chr12:56119229-56122910 | -2.205843475 | 0.808887926 |
| 6283 | chr1:153346183-153348075 | -2.213865542 | 0.800691337 |
| 5641 | chr14:93170151-93215047 | -2.335375403 | 0.818070994 |
| 968 | chr17:7482804-7485429 | -2.37380877 | 0.811727058 |
| 1535 | chr16:88709696-88717457 | -2.390145893 | 0.811221789 |
| 6446 | chr6:134490383-134639196 | -2.474031641 | 0.803899395 |
| 54 | chr19:11670275-11689801 | -2.545227829 | 0.814750654 |
| 972 | chr5:149781199-149792332 | -2.555468419 | 0.834873201 |
| 7305 | chr19:36395302-36399211 | -2.592736985 | 0.814862936 |
| 348 | chr19:45409038-45412650 | -2.819317879 | 0.843791605 |
| n383670 | chr2:89108796-89619842 | -2.833264027 | 0.842163515 |
| n335733 | chr19:45409038-45412650 | -2.954186787 | 0.802094862 |
| 3956 | chr22:38071612-38075809 | -3.048925812 | 0.832298734 |
| 3512 | chr4:71521257-71532348 | -3.136385725 | 0.830766886 |
| 3123 | chr6:32546545-32557562 | -3.20622052 | 0.831143833 |
| 3117 | chr6:32605182-32611429 | -3.731049816 | 0.814141122 |
| 3127 | chr6:32485150-32498006 | -3.76081834 | 0.844970566 |
| n335957 | chr2:89108796-89619842 | -3.774879347 | 0.804196141 |
| 10457 | chr7:23286315-23314729 | -3.858475481 | 0.852597725 |
| 6362 | chr17:34391642-34398841 | -4.111445074 | 0.853287458 |
| n336792 | chr14:106049138-107218968 | -5.265946803 | 0.818070994 |
| n374981 | chr15:77578759-77712556 | -11.59760256 | 0.802038721 |
| n336281 | chr22:22550381-23265153 | -11.64734779 | 0.80690695 |
| n408251 | chr22:21921956-21978323 | -11.81513909 | 0.82163996 |
| XLOC_028290\|- | chr4:57256843-57257059 | -11.89087984 | 0.828569366 |
| n335921 | chr14:106049138-107218968 | -11.90395463 | 0.829652086 |
| n335934 | chr14:106049138-107218968 | -12.17401508 | 0.853086954 |
| 10170 | chr2:169921298-169952677 | -12.23856805 | 0.85830807 |
| n339032 | chr16:2014996-2015617 | -12.78781416 | 0.895826316 |
| 3048 | chr11:5274420-5276011 | -24.37036996 | 0.999863658 |

**Table S2. Primers used in this study.**

| Purpose | Gene/loci | Forward primer (5’-3’) | Reverse primer (5’-3’) | |
| --- | --- | --- | --- | --- |
| qPCR | ***ERF* P1** | AGGGTAAATGTGGTGGTTTGG | AGCATTGTCAGGGGCATCT | |
|  | ***ERF* P2** | GCCTGGCACATCATGAACA | CTCAGGCCTCCTCCTTGG | |
|  | ***RP11-196G18.23*** | GAGGGAGCAGGTTCGCCATA | CTTTGACCGACAGTGTGTTGGG | |
|  | ***GAPDH*-DNA** | GGCTCCCACCTTTCTCATCC | GGCCATCCACAGTCTGG | |
|  | ***GAPDH*-RNA** | GTCGGAGTCAACGGATTTG | TGGGTGGAATCATATTGGAA | |
|  | ***ERF*** | GGCCCTGCGCTATTACTATA | CCAGCCAACCCCACATCAA | |
|  | ***HBG*** | GGTCATTTCACAGAGGAGGACAAG | CCAAAGCTGTCAAAGAACCTCTG | |
| Bisulfite | ***ERF 1st*** | GTATAATGGTAAAGGGTAAATGTGG | CTCAAACCTCCTCCTTAAAAAAAC | |
|  | ***ERF 2nd*** | TTTTGGTTGTAAGATTTAGTGGA | CAAACCTCCTCCTTAAAAAAAC | |
| Overexpression | ***RP11-196G18.23*** | GTCATTTCTTTTCTGTTTTCCTCTCCC | gagattaaacagggcacagaagAGT | |
| Binding sequences deletion | **sgRNA** | CCGCCCGGAGCCAGGAATCG | | CGATTCCTGGCTCCGGGCGG |

**
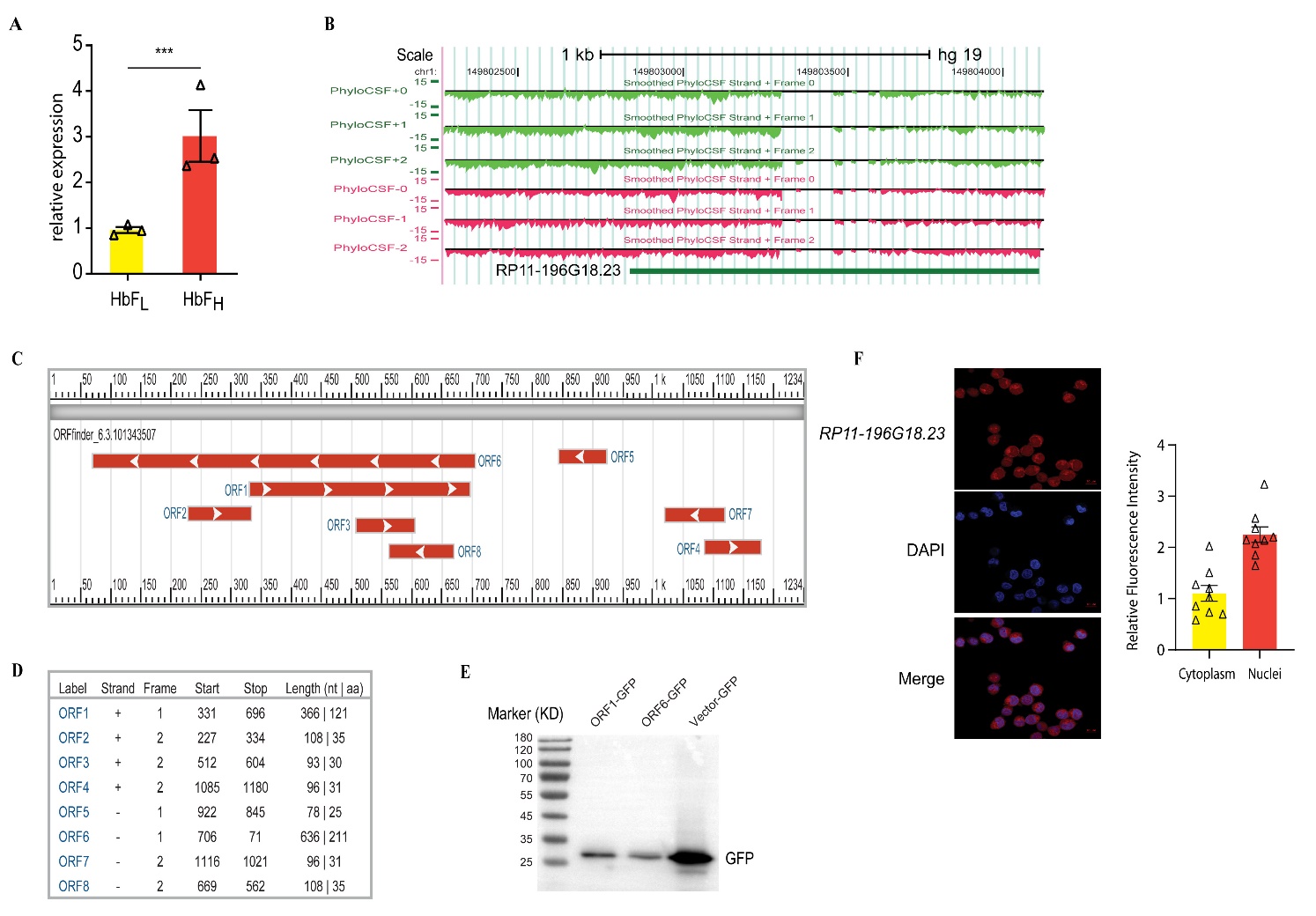
**

**Fig. S1. The screening and characterization of *RP11-196G18.23*.** (**A**) Measurement of *RP11-196G18.23* levels by qPCR in the HbF_H_ and HbF_L_ groups of β^0^-thalassemia patients. (**B-D**) *In* s*ilico* results show the ability of *RP11-196G18.23* to code protein on the basis of Phylogenetic Codon Substitution Frequency (B) and open reading frame (ORF) finder (C-D). Values of phyloCSF below 0 indicate low conservation and low coding ability. The red bars indicate the predicted ORF position. The white arrows indicate the direction of the ORF. (**E**) The coding potential of two largest ORFs (ORF1 and ORF6) was further confirmed by western blotting analysis of fused ORFs with EGFP in the pGEFP-N1 vector transfected into HEK293T cells. **(F)** FISH assay was performed in HUDEP-2 cells. *RP11-196G18.23* sub-localizes to both the nucleus and the cytoplasm. Left**:** fluorescence *in situ* hybridization analysis of *RP11-196G18.23* (red) localization. DAPI (blue) was used to stain nuclei. Right: fluorescence intensity of nuclei relative to the cytoplasm. Data are shown as the mean ± SEM from at least three independent experiments performed in triplicates. ^*^p < 0.05; ^***^p < 0.001.

**
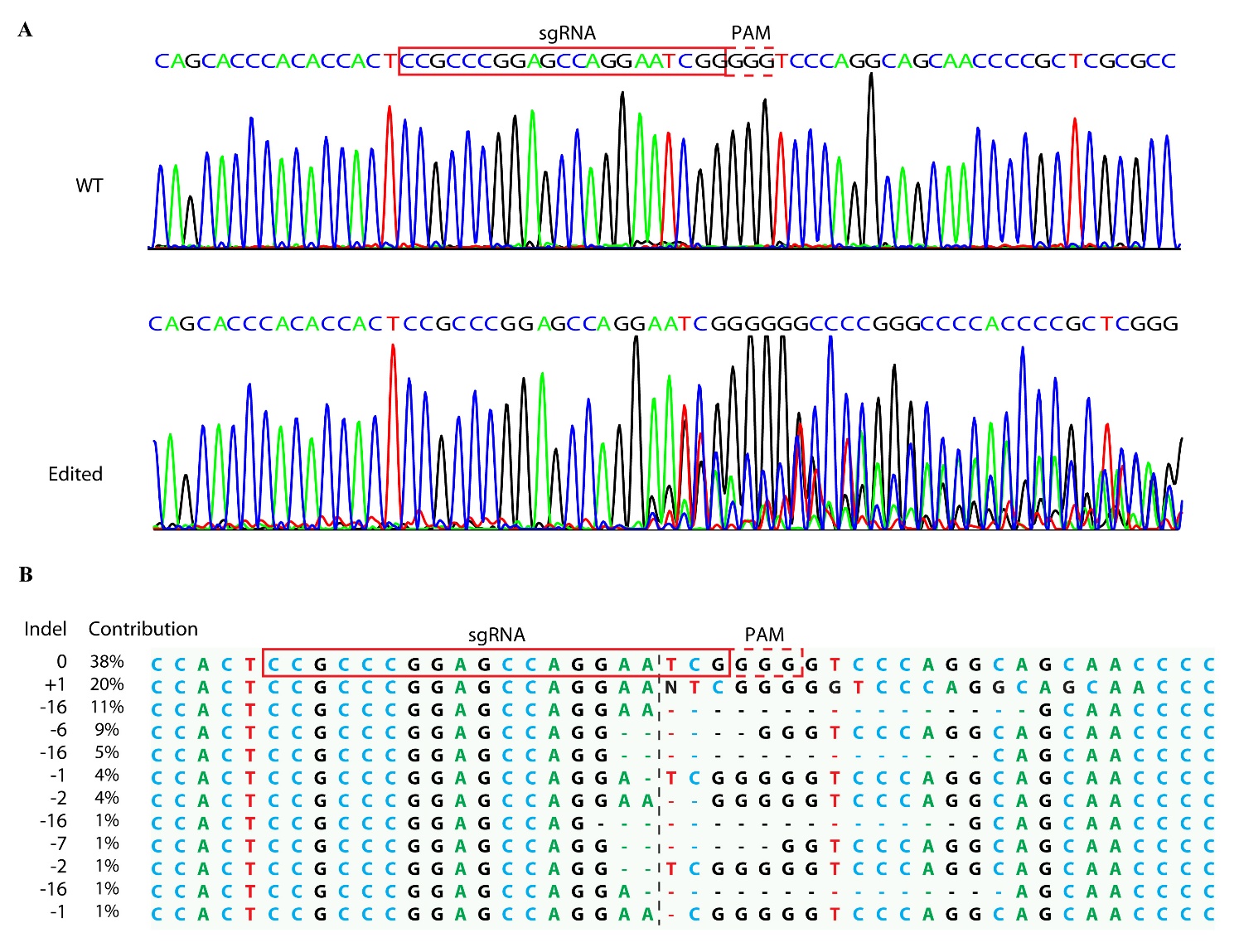
Fig. S2. Indel frequency analysis of disruption the binding sequences of *RP11-196G18.23* on *ERF* promoter.** (A) Representative sequencing chromatographs of PCR products of *ERF* promoter WT and Edited HUDEP-2 cells. (B) The indel distribution pattern of *ERF* promoter edited HUDEP-2 cells.


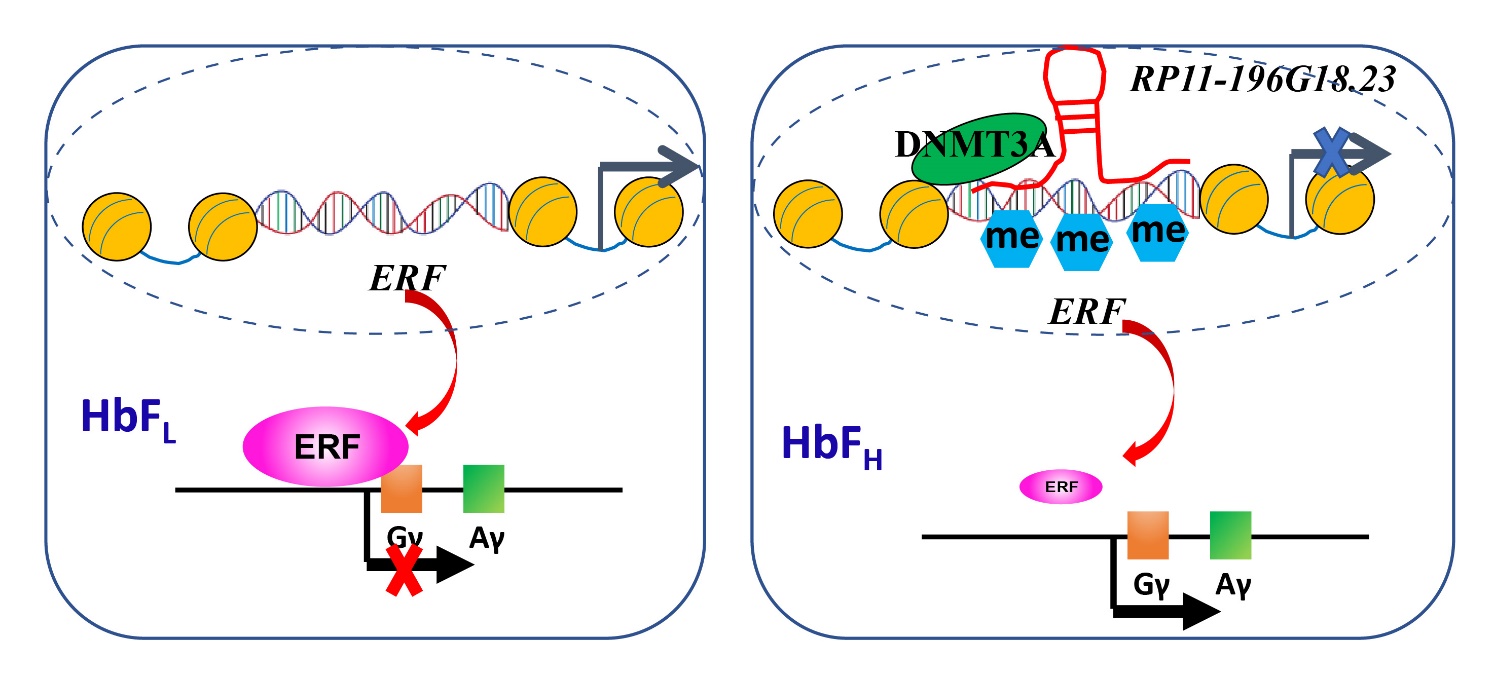


**Figure S3. The proposed model for γ-globin reactivation mediated by the *RP11-196G18.23*.** Without methylation, ERF was highly expressed and bound to γ-globin gene promoter to inhibit its expression. After RP11-196G18.23 bound to ERF promoter, DNMT3A was recruited and mediated hypermethylation in ERF promoter, resulting in downregulation of ERF and reactivation of γ-globin.
